# High Fat Diet, Metabolic Syndrome and Systemic Lupus Erythematosus: A Causal Loop

**DOI:** 10.31138/mjr.31.2.172

**Published:** 2020-06-15

**Authors:** Carlo Perricone, Fulvia Ceccarelli

**Affiliations:** 1 Reumatologia, Dipartimento di Medicina, University of Perugia, Italy; 2 Lupus Clinic, Dipartimento di Medicina Interna e Specialità Mediche, Sapienza University of Rome, Rome, Italy

**Keywords:** Systemic lupus erythematosus, TLR, TLR7, diet, metabolic syndrome, inflammation, obesity, insulin resistance, cytokines

Mechanisms of systemic lupus erythematosus (SLE) exacerbation are still unclear. Several lines of evidence pointed out that metabolic syndrome is a frequent manifestation in SLE patients, with approximately 35% being overweight and another 27% obese.^1^ Obesity is, per se, a proinflammatory status in which there is an increased gene and protein expression of proinflammatory molecules such as IL-17, IL-23 and TNF-α.^2^ In childhood-onset SLE, TNF-α levels are associated with obesity and body fat content. It has been observed that obese lupus patients tend to have a have a worsening of their chronic inflammatory status, probably because of increased oxidative stress, increased C-reactive protein expression and elevated protein oxidation and lipid hydroperoxidation.^3^ The metabolic syndrome (MetS) is recognized as a chronic proinflammatory and prothrombotic state that aggravates insulin resistance, oxidative injury, and cardiovascular risk. It includes several risk factors such as central obesity, insulin resistance, dyslipidaemia, increased blood pressure, and endothelial dysfunction.^4^ The tissue possibly responsible for these events is the white adipose tissue that becomes dysfunctional and produces excessive amounts of pro-inflammatory soluble mediators including interleukin 6 (IL-6), and adipokines such as leptin, adiponectin, and resistin.^5^

Toll-like receptors (TLRs) have been recently emerged as main actors in this pantomime playing a role linking inflammation, obesity and insulin resistance, both in humans and in murine models.^6^ Among TLRs, some have been specifically evaluated in the context of obesity, such as TLR2, TLR6, and TLR7 that showed a lower expression after a high fat diet in the adipose tissue.^7^

TLR7 gained attention also because it was shown to promote the effector functions of purified CD8+ T cells in vitro and the upregulation of glucose uptake and glycolysis.^8^

Wong et al. in an extensive review on the argument, underlined how metabolic syndrome, TLR expression and inflammation are linked in a way that MetS triggers TLR activation via several mechanisms including AGEs, FFAs, HMGB1, HSPs, and endotoxins, and TLR activation in turn leads to inflammation via MyD88 and TRIF dependent pathways.^9^

Recently, Hanna Kazazian N. and colleagues described in their paper a possible implication of TLR7 signalling in high-fat diet (HFD)-induced metabolic syndrome and exacerbation of lupus autoimmunity in a mouse model of lupus, the TLR8-deficient (TLR8ko) mice.^10^ This mouse spontaneously develops lupus-like disease due to increased TLR7 signalling by dendritic cells (DCs). Those mice fed with a HFD had an aggravated lupus condition characterized by increased overall immune activation, anti-DNA autoantibody production, and IgG/IgM glomerular deposition that were coupled with increased kidney histopathology.

Also, liver inflammation was worsened by HFD which may explain at least partly the relatively frequent sub-clinical liver inflammation that can be found in patients with SLE. T cells seem to be the major driver of such phenomena; in particular, HFD led to a reduction of splenic CD4+ and CD8+ T cells both in WT and TLR8ko mice, compared to standard-diet (SD)-fed mice, while the authors did not observe any difference between SD- and HFD-fed TLR7/8ko mice, concluding that both in the spleen and liver, HFD leads to an increase of effector memory and decrease of naïve CD4+ and CD8+ T cells in a TLR7-dependent manner. In conclusion, it seemed that upon HFD, TLR7/8ko mice did not develop SLE and both TLR7ko, and TLR7/8ko mice were fully protected from metabolic abnormalities, including body weight gain, insulin resistance, and liver inflammation. All these data together may suggest that TLR7 might be a novel approach as a tailored therapy in SLE and metabolic diseases.

It is noteworthy in this context that a very recent paper linked the interferon pathway with increased adipose tissue and metabolic inflammation. These authors found that adipose IRF5 gene and protein expression was up-regulated in obese compared with lean individuals and IRF5 gene expression was associated positively with adipose inflammatory signatures including local expression of TNF-α, IL-6, CXCL8, CCL-2/5, IL-1β, IL-18, CXCL-9/10, CCL7, CCR-1/2/5, IRF3, MyD88, IRAK-1, and inflammatory macrophage markers and, importantly, also with TLR-2/7/8/9.^11^

Thus, the question is whether SLE can be improved through diet, including calories restriction or microbiota modification. Mouse models of SLE have proved so,^12^ and the improvement goes together with a lower atherogenic risk due to reduced expression of IL-12 and IFN-γ, reduced NF-κB activation, and the induction of T regulatory cells.^13^ It is likely that a tailored diet, possibly based on the genetic study of the affected individual, may improve through TLR7, the outcome of lupus disease.
